# C-kit induces epithelial-mesenchymal transition and contributes to salivary adenoid cystic cancer progression

**DOI:** 10.18632/oncotarget.1606

**Published:** 2014-01-06

**Authors:** Ya-ling Tang, Yun-long Fan, Jian Jiang, Kai-de Li, Min Zheng, Wei Chen, Xiang-rui Ma, Ning Geng, Qian-ming Chen, Yu Chen, Xin-hua Liang

**Affiliations:** ^1^ State Key Laboratory of Oral Diseases West China Hospital of Stomatology (Sichuan University), Chengdu Sichuan, People's Republic of China; ^2^ Department of Oral Pathology, West China Hospital of Stomatology (Sichuan University), Chengdu Sichuan, People's Republic of China; ^3^ Department of Oral and Maxillofacial Surgery, West China Hospital of Stomatology (Sichuan University), Chengdu Sichuan, People's Republic of China

**Keywords:** salivary adenoid cystic cancer, epithelial–mesenchymal transition (EMT), c-kit, cancer stem cell, metastasis

## Abstract

Epithelial–mesenchymal transition (EMT) is associated with salivary adenoid cystic cancer (ACC) progression and metastasis. Here, we report that ectopic overexpression of c-kit in ACC cell lines is sufficient for acquisition of mesenchymal traits, enhanced cell invasion, along with stem cell properties defined by the presence of a CD133 + /CD44 + cell subpopulation. c-kit positively regulated expression of known EMT inducers, also activating TGF-β to contribute to EMT. c-kit itself was induced by TGF-β in ACC cell lines and required for TGF-β–induced EMT. Xenograft experiments showed that c-kit cooperated with oncogenic Ras to promote tumorigenesis in vivo. Finally, in human specimens of ACC, we found that c-kit was abnormally overexpressed and correlated with the prognosis of ACC. Our findings define an important function for c-kit in ACC progression by orchestrating EMT, and they implicate this gene product as a marker of poor prognosis in this disease.

## INTRODUCTION

Adenoid cystic carcinoma (ACC), composed of luminal epithelial cells, abluminal myoepithelial cells, and stromal cells, is the second most common malignant salivary gland cancer accounting for 10-25% of patients[1]. ACC is characterized by insidious invasion into adjacent tissue and hematogenous spread to distant organs (lung, bone and liver) and patients with distant metastasis have a 5-year survival rate as low as 20%[2]. Epithelial-mesenchymal transitions (EMT), interconverting epithelial cell types into cells with mesenchymal attributes, plays a pivotal role in various steps of tumor metastatic cascades [3, 4]. Biomarkers of EMT such as Snail1 and Slug have emerged as being associated with increased tumor aggressiveness and may be useful in diagnosing ACC [5, 6]. Therefore, EMT may also be a necessary, but not sufficient, step of ACC metastasis as exemplified in breast cancer and lung cancer and lots of work should be done [7].

EMT program can be choreographed by a number of transcription factors to initiate the complex transcriptional program to impart malignant traits to cancer cells such as Snail1, Slug, ZEB1, ZEB2, Prrx1, HOXB7[8]. In addition, an EMT program can also be induced by several signal pathways, including TGF-β, NF-κB, TNF-α, Wnt, Notch, and Hedgehog [9, 10]; among them, the TGF-β signaling plays a significant role in contributing to the initiation of EMT [11]. Recently, EMT has been shown to confer a cancer stem cell (CSC) phenotype to contribute to the tumor malignant progression [12, 13]. However, the link of EMT and CSC in ACC is needed to be investigated.

c-kit, CD117, is the transmembrane tyrosine kinase receptor, and stem cell factor (SCF) is its primary ligand [14]. SCF/c-kit signaling pathway has been previously implicated in normal hematopoiesis, melanogenesis, and gametogenesis through the formation and migration of c-kit(+) cells. Besides functioning as a transcriptional factor in regulation of development processes, binding of SCF to c-kit can trigger pathways involved in the maintenance of progenitor cells[15]. c-kit has been regarded as a cancer stem cell marker as Oct4, Nanog, SOX2, CD133, CD44, ALDH1, Gata-4, Isl-1, and nestin in primary non small cell lung cancer, ovarian cancer and hepatocellular carcinoma[16]. Recently, this cell surface receptor has been shown to commonly isolate progenitor cells of submandibular glands and overlaps with expression of other commonly known stem cell markers (e.g. Nanog, Oct3/4) [17]. As a marker of stem cells in normal salivary gland, c-kit could also be a potential marker for CSC in ACC as well and play an important role in the maintenance of stem cell properties[2].

Both TGF-β and Wnt signal pathways have been implicated in cancer progression and EMT [18]. TGF-β1 has been shown to highly express in ACC patient samples and SACC-LM cell line and induced Smad2 phosphorylation and promoted the migration and invasion of SACC-83 cells [19]. And imatinib mesylate, a clinical drug that blocks c-kit kinase activity, suppressed TGF-β1 expression in CD90 (+) cells as well as CD90 (+) cell-induced motility of EpCAM (+) cells[20]. Moreover, studies into the mechanisms of c-kit in ovarian neoplastic processes suggest an important role for the activation of Wnt/β-catenin and ATP-binding cassette G2 downstream of c-kit [21]. Therefore, based on our previous results that c-kit has been demonstrated in ACC and plays a critical role in tumor invasion, metastasis, and decreased survival [22], here we speculate that c-kit might be a potential marker for CSC in ACC and play a role in ACC progression and aggression through induction of an oncogenic EMT.

In this study, we showed that c-kit was able to activate EMT program in ACC cells, increase the number of CD133^+^/CD44^+^ population and potentiate mammosphere-forming ability. We also showed that c-kit cooperated with the activated oncogenic Ras to endow the cells with the more capacity of the tumorigenicity in vivo. We further showed that human ACC tissue has increased c-kit expression. These data implicate a novel role of c-kit in inducing EMT and the link of EMT and CSC, and its close association with the poorer prognosis of human ACC.

## RESULTS

### Exogenous c-kit expression induced EMT in human salivary adenoid cystic cancer cells

**Figure 1:**
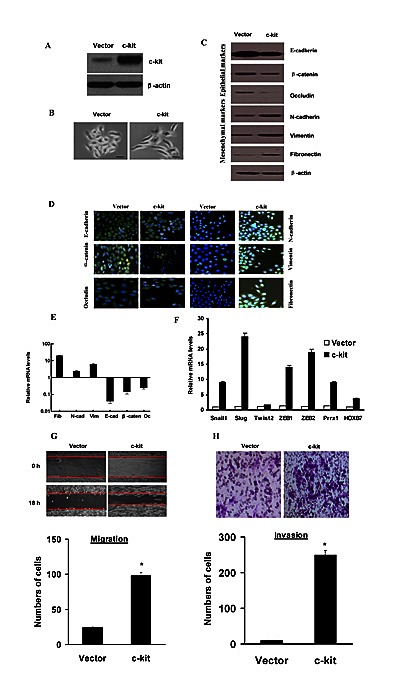
Ectopic expression of c-kit in ACC-M cells induced an EMT program A), Immunoblotting assessment of the ectopic c-kit protein expression after plasmid transfection in ACC-M cells. B), Morphologic change of ACC-M cells expressing c-kit or empty vector. Scale bar, 100 mm. C), Immunoblotting analysis of expression of the epithelial markers E-cadherin, β-catenin, and Occludin, and the mesenchymal markers Fibronectin, Vimentin, and N-cadherin. D), Immunofluorescence staining for the epithelial and mesenchymal markers. Scale bar, 100 mm. E), The expression of E-cadherin, β-catenin, Occludin, fibronectin, Vimentin, and N-cadherin mRNAs were assessed by real-time PCR. F), mRNA expression levels of known EMT inducers were assessed by real-time PCR. Error bars represent the mean±SD of triplicate experiments. G and H), Migration (G) and invasion (H) assays in stable ACC-M cells. The mean was derived from cell counts of 5 fields, and each experiment was repeated 3 times (* *P* < 0.001, compared with the control). Representative images of migrated and invaded cells are shown.

To investigate the role of c-kit in EMT, we stably overexpressed c-kit in the human salivary adenoid cystic cancer cell line ACC-M, as confirmed by immunoblotting (Figure 1A) and real-time PCR (Figure S1). We observed that ACC-M transfected with vector retained their cobblestone-like morphology with tight cell–cell adhesion, whereas cells expressing exogenous c-kit displayed an elongated fibroblast-like morphology with scattered distribution in culture (Figure 1B). We then examined both epithelial and mesenchymal markers by immunoblotting (Figure 1C) and immunofluorescence (Figure 1D). As can be seen, the c-kit-expressing ACC-M cells exhibited a significant down-regulation of β-catenin and E-cadherin and complete loss of Occludin from cell–cell contacts; meanwhile the mesenchymal markers Fibronectin, Vimentin, and N-cadherin were dramatically up-regulated. Real-time PCR analyses also revealed the mRNA expression of E-cadherin, β-catenin and Occludin, and concomitant induction of N-cadherin, Fibronectin, and Vimentin mRNAs in c-kit-expressing ACC-M cells (Figure 1E). These morphologic and molecular changes suggested an apparent transition of the c-kit-expressing ACC-M cells from an epithelial to mesenchymal status. To further probe the possible interactions between c-kit and other EMT-inducing transcription factors, we examined the expression of other known EMT inducers. We showed that the endogenous mRNA levels of Slug, Snail1, ZEB1, ZEB2, Prrx1, HOXB7 were elevated in response to c-kit overexpression, to a variable extent, whereas the Twist2, mRNA level exhibited no detectable change (Figure 1F). We also ectopically overexpressed other EMT inducers Snail1 and Slug in ACC-M cells. We observed that Snail1 and Slug induced EMT in ACC-M cells, and up-regulated c-kit mRNA expression in ACC-M cells (Figure S2). Typically, the EMT phenotype is usually accompanied by the acquisition of cell traits such as greater migration and more invasive ability. As shown in Figure 1G and H, c-kit-expressing ACC-M cells dramatically increased their migratory and invasive behaviors. Similar results were observed in ACC-2 cells, a prototypic cell model for ACC study (Data not shown). Together, these results show that c-kit is a novel inducer of EMT and it promotes cell migration and invasion in ACC-2 and ACC-M cells.

We next carried out a loss-of-function assay to further study the role of c-kit in ACC-M and ACC-2 cells. The effect of c-kit silencing on the invasive ability of ACC-M and ACC-2 was investigated using transwell invasion assays. The results indicated that c-kit knockdown prominently impaired the migration and invasion ability of ACC-M and ACC-2 cells, and it reduced cell proliferation (Figure S3A-H). Moreover, suppression of c-kit expression resulted in E-cadherin up-regulation and N-cadherin down-regulation. However, no detectable changes were observed in other EMT markers (data not shown). And down-regulation of c-kit did not cause significant cellular morphologic changes in ACC-M and ACC-2 cells. Thus, our data suggested that the suppression of c-kit could partially reverse the EMT phenotype of ACC-M and ACC-2 cells. In addition, we treated ACC cell lines with 5μM Gleevec (Imatinib mesylate), the inhibitor of c-kit [23].The result showed that Gleevec had reversal of the enhanced migration and invasion ability of ACC-M (Figure S4) and ACC-2 cells with c-kit overexpression, suggesting that c-kit is required for the EMT and the migration and invasion of ACC cell lines, and targeting c-kit could be a promising therapeutic strategy for ACC metastasis.

Loss of E-cadherin has been regarded as a critical event in EMT. We next carried out a luciferase reporter assay to investigate whether c-kit could transcriptionally regulate the E-cadherin expression. The results showed that the relative luciferase activity of E-cadherin was not changed in 293T cells transiently cotransfected with E-cadherin promoter reporter together with c-kit expression construct or with empty vector (Figure S5A), indicating that c-kit may indirectly regulate E-cadherin transcription. In line with these observations, c-kit increased the expression of known EMT inducers (including Slug and Snail1) that act to directly repress E-cadherin transcription, during c-kit-induced EMT (Figure 1F). To test the effect of c-kit on the transcription activity of promoters of these 2 EMT inducers, we transiently transfected 293T cells with luciferase reporter constructs containing proximal promoter of Snail1 or Slug. We found that the relative luciferase activity of Slug was increased proportionally to the increasing amount of c-kit in 293T cells (Figure S5B). However, the Snail1 promoter activity was not changed (Figure S5C). These findings suggest that E-cadherin transcriptional inactivation during c-kit -induced EMT may be a result of induction of Slug expression, a well known upstream repressor of E-cadherin.

### c-kit-mediated EMT generated stem cell-like cells

**Figure 2:**
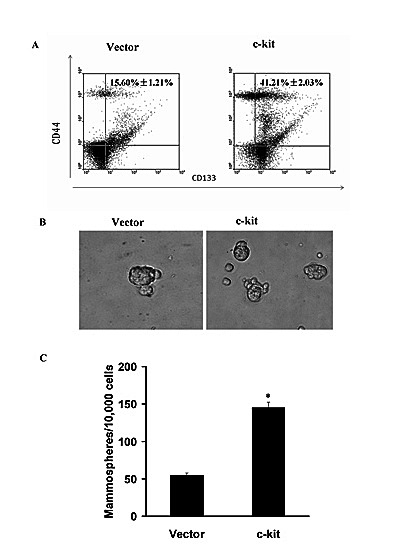
c-kit-induced EMT generated stem cell-like cells A), FACS analysis of cell-surface markers CD133 and CD44 in ACC-M cells expressing c-kit or empty vector. Percentages of mean CD133^+^/CD44+ subpopulation±SD based on triplicate experiments are indicated. B), Phase contrast images of mammospheres formation. Scale bar, 100 mm. C), Quantification of mammosphere numbers formed from 3 independent experiments (error bar, mean±SD; * *P* < 0.05, compared with the control)

EMT has been considered to be accompanied by the acquisition of the cancer stem cell properties, including tumorigenicity, ability to redifferentiate into an epithelial tumor, and ability to form spheroids [24]. CD133 and CD44 has been regarded a cancer stem-like cell marker and CD133- or CD44-positive cells play an important role in morphogenesis of ACC[25]. To determine whether c-kit has the effect to lead to the stem cell phenotypes upon induction of EMT, we carried out Fluorescence Activating Cell Sorter (FACS) to identify CD133-positvie or CD44-positive populations. We observed that the c-kit-expressing ACC-M cells exhibited a significant increase in the CD133^+^/CD44^+^ stem cell population compared with the control cells (Figure 2A). Meanwhile, as evidenced in Figure 2B and C, the c-kit-expressing ACC-M cells increased both in size and in number of mammospheres in comparison with the control cells. We thus concluded that the c-kit-induced EMT generates mesenchymal cells with stem cell-like phenotypes, a feature recently defined for EMT inducers.

### c-kit cooperated with activated oncogenic Ras to promote tumorigenesis

**Figure 3:**
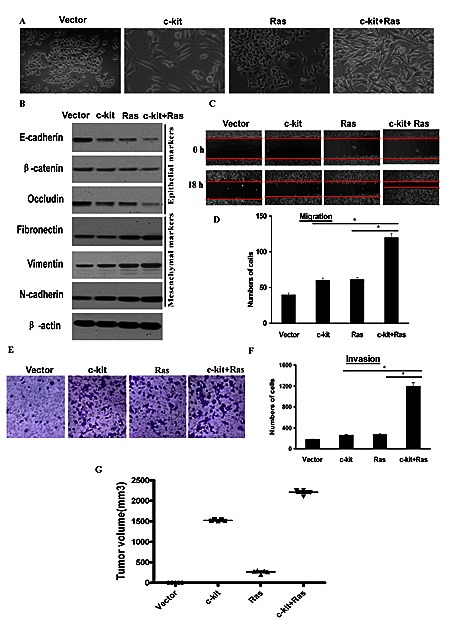
c-kit cooperated with activated oncogenic Ras to promote tumorigenesis ACC-M cells were infected with empty vector, retroviral vector encoding -kit, or sequentially infected with retroviral vector encoding H-RasV12G (Ras) and c-kit as indicated. A), Phase contrast images of the cell morphology. Scale bar, 100 mm. B), Immunoblotting analysis of expression of the epithelial and mesenchymal markers. C and D,E,F), Migration (C and D) and invasion (E and F) assays. The mean was derived from cell counts of 5 fields, and each experiment was repeated 3 times. Representative images of migrated or invaded cells are also shown. G), Individual tumor volume was measured according to the formula: Л/6 × length ×width^2^ at the 7^th^ week after injection.

It has recently been reported that EMT inducers Twist1, Twist2, and Slug cooperate with activated oncogenic Ras to promote even more dramatic characteristics of EMT [26]. To investigate this possible cooperation, we coexpressed c-kit with oncogenic H-RasV12G in ACC-M cells. As shown in Figure 3A, coexpression of c-kit and oncogenic Ras led to a more dramatic morphologic change, characterized by prominently elongated spindle-shaped cells. In addition, coexpression of both c-kit and H-RasV12G triggered a further reduction of E-cadherin, β-catenin, and Occludin relative to either c-kit or H-RasV12G alone; and an increasement of Fibronectin, Vimentin and N-cadherin (Figure 3B). Furthermore, ACC-M cells expressing c-kit+H-RasV12G exhibited higher migration (Figure 3C, D) and invasive (Figure 3E, F) ability than cells expressing either c-kit or H-RasV12G alone. Meanwhile, all these cells exhibited roughly the same growth rates with no statistically significant differences (Figure S6). These data implicated that c-kit and H-RasV12G worked synergistically in inducing a more prominent EMT phenotype.

To further establish whether c-kit is able to trigger tumorigenesis in vivo, we tested its effect in a xenograft mouse model. We detected the primary tumor growth 6 weeks after the nude mice were subcutaneously injected with ACC-M cells stably expressing c-kit alone. Meanwhile, ACC-M cells expressing oncogenic H-RasV12G alone produced small palpable tumors. Interestingly, coexpression of both c-kit and H-RasV12G resulted in rapid development of very large tumors (Figure 3G). These results clearly indicate that c-kit and oncogenic H-RasV12G synergistically work to induce tumorigenesis in vivo.

### High expression of c-kit and Slug was correlated with the poor prognosis of salivary adenoid cystic cancer

**Figure 4:**
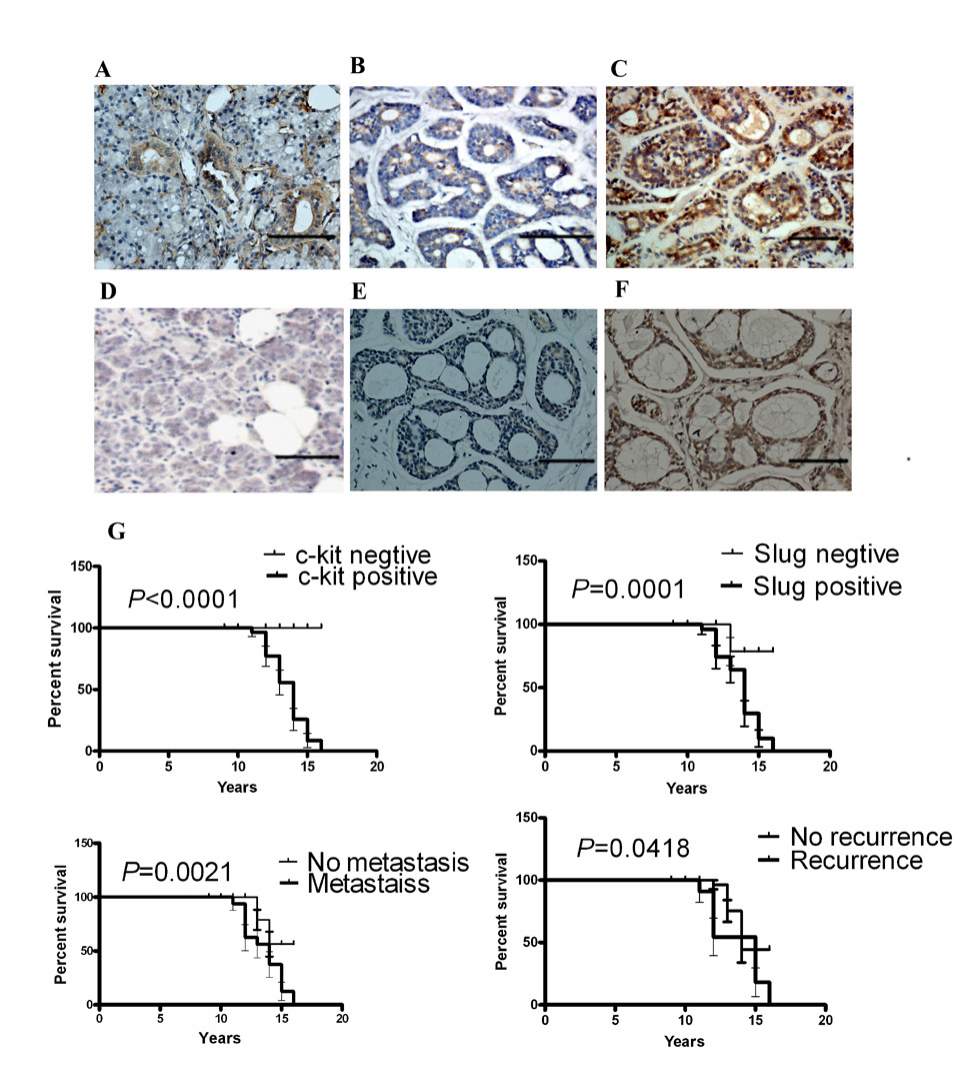
c-kit and Slug expression was associated with invasive subtypes of human ACC Representative images of the immunohistochemical staining of c-kit and Slug in ACC samples. A), c-kit in normal human salivary tissue. B), c-kit in weak tumor staining. C), c-kit in strong tumor staining. D), Slug in normal human salivary tissue. E), Slug in weak tumor staining. F), Slug in strong tumor staining. Scale bar, 100 mm. G), Kaplan-Meier survival analysis in patients with ACC. Overexpression of c-kit and Slug in ACC was associated with a shorter overall survival in the respective group.

To evaluate the clinical relevance of c-kit and Slug expression, we carried out immunohistochemistry staining of c-kit and Slug in 121 human salivary adenoid cystic carcinoma samples representing different subtypes and several normal human salivary gland tissues. There were 108/121(89.26%) cases of positive immunoreaction for c-kit in ACC (Figure 4A,B,C). 87/121(71.90%) cases of ACC yielded a positive immunoreaction for Slug(Figure 4D, E, F). c-kit and Slug expression were significantly associated with tumor site, TNM stage, histological pattern, perineural invasion, local regional recurrence and distant metastasis. However, there was no significant association of the c-kit and Slug expression status with age, sex, complaints, and resection margins of patients. And, a significant association between the positive expression of c-kit and that of Slug was observed (*P* =0.046), 81 of the samples with positive Slug expression also exhibited positive c-kit expression (These results has been reportedly in Oral Oncology by our group).

Moreover, survival curves were computed with the Kaplan-Meier method and compared between groups by using the log-rank test. The patients with positive c-kit or Slug had a poorer prognosis (a lower survival rate) than those with negative (*P* <0.0001; *P*= 0.0001, respectively). The patients with metastasis or recurrence had a poorer prognosis (a lower survival rate) than those without (*P* =0.0021; *P* =0.0418, respectively) (Figure 4G). These observations implicate the potential usefulness of the aberrant high c-kit and Slug expression as a novel prognostic molecular marker for ACC.

### Activation of TGF-β1 was necessary for c-kit-induced cell motility

**Figure 5:**
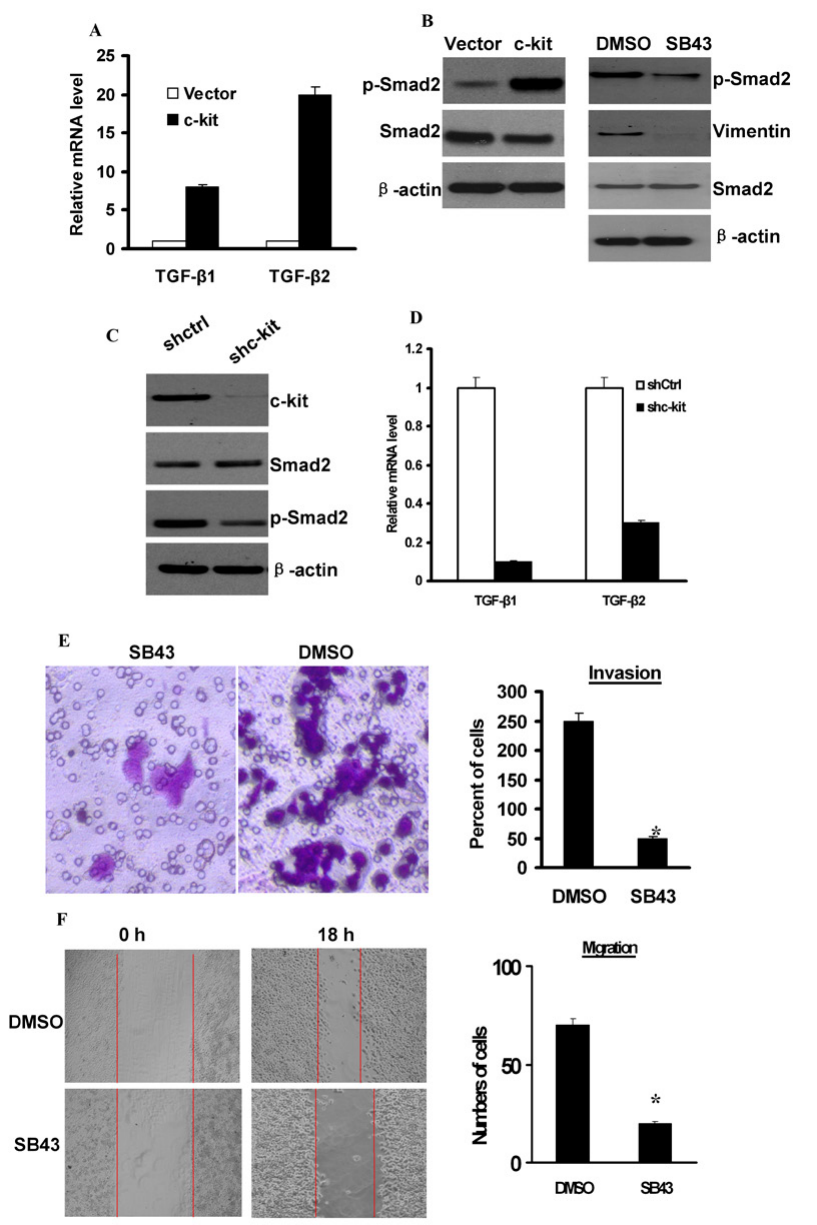
Activation of TGF-β1 was necessary for c-kit-induced cell motility A), Expression of TGF-β1 and TGF-β2 mRNAs was determined by real-time PCR in ACC-M cells expressing empty vector or c-kit. B), left, Immunoblots of p-Smad2 and Smad2 protein. Right, c-kit-ACC-M cells were treated with 10 mmol/L SB431542 (SB43) for 24 hours. Immunoblots of p-Smad2, Smad2 protein, and Vimentin. C), c-kit-ACC-M cells were stably infected with c-kit shRNA or non-target vector shRNA (shCtrl). Immunoblotting of c-kit, p-Smad2, and Smad2 protein. D), Real-time PCR analysis of the expression of TGF-β1 and TGF-β2 mRNAs. E and F), Migration (E) and invasion (F) assays of SB431542-treated c-kit-ACC-M cells. The mean was calculated from cell counts of 5 fields, and each experiment was repeated 3 times (* *P* < 0.001, compared with the DMSO treated). Representative images of migrated or invaded cells are shown.

Several lines of evidence have implicated the involvement of TGF-β in EMT process both in cancer progression [27]. We next intended to identify whether TGF-β signaling is activated in c-kit-induced EMT. Our real-time PCR revealed an increased expression of TGF-β1 and TGF-β2 mRNAs in c-kit-expressing ACC-M cells (Figure 5A). In addition, the level of phosphorylated Smad2 protein, a downstream effector of TGF-β pathway, was significantly increased in c-kit-expressing ACC-M cells (Figure 5B left). Moreover, c-kit silencing efficiently decreased the level of phosphorylated Smad2 and the expression of TGF-β1 and TGF-β2 mRNAs in c-kit-expressing ACC-M cells (Figure 5C, D); whereas knockdown of c-kit upregulated E-cadherin expression and downregulated N-cadherin expression. Apparently, these experiments pointed to a reinforced TGF-β signaling upon ectopic c-kit expression in ACC-M cells. To further validate that TGF-β signaling is responsible for the c-kit-induced EMT and the enhanced cell motility, we used a specific TGF-β receptor kinase inhibitor SB431542 to block the TGF-β signaling in c-kit-expressing ACC-M cells. We found that suppression of TGF-β signaling by the inhibitor reduced Smad2 phosphorylation level and down-regulated Vimentin expression (Figure 5B right), without affecting the morphologic and molecular features of c-kit-expressing ACC-M cells undergoing EMT. Moreover, treatment of c-kit-expressing ACC-M cells with SB431542 reduced their migration and invasive ability (Figure 5E and F). These results suggest that the intensified TGF-β signaling induced by c-kit promotes cell motility, and this partly contributes to EMT.

**Figure 6:**
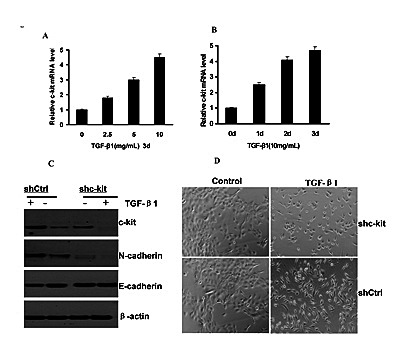
c-kit was necessary for TGF-β–induced EMT A and B), c-kit mRNA expression levels in ACC-M cells treated with activated TGF-β1 at indicated time and concentrations. Error bar represents mean±SD of triplicate assays. C), Immunoblotting of c-kit, E-cadherin, and N-cadherin after 48-hour treatment of TGF-β1(10 ng/mL) in ACC-M cells with shCtrl and shc-kit. D), morphology of ACC-M cells with shCtrl and shc-kit after TGF-β1 treatment as in C. Scale bar, 100 mm.

TGF-β signaling has been shown to be able to induce EMT in human salivary adenoid cystic carcinoma and hepatocellular carcinoma[19, 20]. Here, we showed that c-kit mRNA was also induced in ACC-M (Figure 6A and B) and ACC-2(Figure S7) cells, in a dose- and time-dependent manner upon the addition of TGF-β1 to the cell culture medium. To investigate whether c-kit is required for TGF-β–induced EMT, we knocked down c-kit expression in ACC-M cells with short hairpin RNA (shRNA) and examined their responses to TGF-β1 treatments. ACC-M cells expressing c-kit shRNA (ACC-M-shRNA c-kit) or non-target control shRNA (ACC-M-shRNA-neg) were treated with TGF-β1.We observed that c-kit expression was induced 48 hours after TGF-β1 addition in the ACC-M-control shRNA cells, whereas ACC-M-shRNA c-kit cells exhibited a reduction in the basal expression level of c-kit as well as in the induction of c-kit after TGF-β1 stimulation (Figure 6C). We also found that TGF-β1 treatments induced EMT in ACC-M–shCtrl cells, but not in ACC-M–shRNA c-kit cells (Figure 6D). Consistent with morphologic changes, N-cadherin expression was increased and E-cadherin expression was decreased in ACC-M–shCtrl cells after TGF-β1 treatments. However, under the same conditions E-cadherin and N-cadherin expression were not changed in ACC-M–shRNA c-kit cells (Figure 6C). These experiments suggest that c-kit is also involved in and required for TGF-β–induced EMT.

## DISCUSSION

Increasing evidences suggest that c-kit overexpression is associated with several human cancers including colorectal carcinoma, breast cancer, and ACC [28]. c-kit has been regarded as a cancer stem cell marker in primary non-small cell lung cancer, ovarian cancer and hepatocellular carcinoma[16, 17]. EMT can confer a cancer stem cell phenotype to contribute to the tumor malignant progression. Thus, we hypothesize that c-kit may link EMT and CSC and play an important role in ACC. We find in this study that c-kit functions as a trigger for EMT to contribute to ACC progression, a previously unreported role of c-kit in ACC. Specifically, our data show that c-kit represses the epithelial phenotype, induces the mesenchymal phenotype, and c-kit-mediated EMT generates stem cell-like cells.

Recently, it has been reported that the concurrent inhibition of PI3K and MAPK signaling is required to suppress oncogenic c-kit activity in c-kit mutant melanomas [29]. SCF was required for migration and invasion of human colon carcinoma cells DLD-1 through reconstituted basement membranes and up-regulated matrix metalloproteinase (MMP)-9 activity[30]. Moreover, miR-221 suppresses colorectal cancer metastasis and invasion by down-regulating c-kit Stat5A and ETS1 [31]. Accumulating evidence indicates that EMT inducers such as Snail1 and Slug contribute to tumor invasion and metastasis [3, 4]. These results suggest that c-kit may act in a similar fashion as Snail1 and Slug, orchestrating an EMT in tumor progression.

Moreover, we have shown in this work that c-kit up-regulates the mRNA expression of several EMT-inducing transcription factors and c-kit expression is induced by a large number of known regulators of the EMT program, notably the Snail1, Slug and TGF-β1. This is in accordance with the discovery that overexpression of one of the EMT inducers up-regulates a subset of other EMT-inducing transcription factors, implicating the interactions among these EMT inducers [32]. Loss of E-cadherin is considered a hallmark of EMT [3,4]. Our results show that c-kit may indirectly down-regulate the transcription of E-cadherin. It is reported that EMT inducers such as Snail1, Slug, ZEB1, ZEB2 are able to transcriptionally repress E-cadherin expression either directly or indirectly [33]. Casas et al [34] showed that Twist1 indirectly suppresses E-cadherin transcription to promote EMT through directly binding to the Snail2(Slug) gene promoter to activate its transcription. We show in this study that c-kit indirectly down-regulates the expression of E-cadherin through activating Slug expression, suggesting Slug may be a molecular target in c-kit inducing EMT. This data is in line with the following previous results. Catalano et al [35] showed that induction of Slug by autocrine production of SCF and c-kit activation is strongly resistant to conventional chemotherapy and plays a key role in conferring a broad spectrum chemoresistance in malignant mesothelioma patients. In radiation-induced destruction, Pérez-Losada et al [36, 37] reported that Slug has been identified as the molecular target that mediates the radioprotection through SCF/c-kit, and Slug may contribute to the biologic specificity to the SCF/c-kit signaling pathway in stem cell mobilization.

More importantly, our data have suggested that c-kit is also involved in and required for TGF-β–induced EMT. We show in our study that the autocrine TGF-β signaling is activated in c-kit-induced EMT; meanwhile TGF-β signaling induces c-kit expression in ACC cells and knockdown of c-kit blocks TGF-β–induced EMT. Thus, we speculate that the c-kit-TGFβ-c-kit feedback loop presumably functions as a novel signaling pathway in EMT regulation, as well as in ACC progression. A recent study by Scheel et al[38] showed that TGF-β signaling pathway and both the canonical and noncanonical Wnt signaling are activated in EMT program induced by diverse stimuli, and they propose that these signalings further maintain the EMT, and disruption of these extracellular autocrine signaling abrogates EMT. In support of the involvement of additional EMT-promoting pathways, it has been documented that c-kit activates the Wnt/β-catenin and ATP-binding cassette G2 signaling to mediate chemoresistance and tumor-initiating capacity of ovarian cancer cells [21]. These results further suggest that c-kit function as a trigger for EMT to contribute to cancer progression.

CD133 or CD44 has been thought to represent a promising CSC marker and CD133- or CD44-positive cells play an important role in morphogenesis of salivary ACC [25,39]. In this study, we have shown that ectopic expression of c-kit in ACC-M/2 cells increases the CD133^+^/CD44^+^ subpopulation and enhances the mammosphere forming ability, a property of the stem cells. This result is in line with the discovery that EMT can induce non-cancer stem cells to acquire CSC-like properties [24]. This supports that exogenous c-kit is an EMT- inducer and contribute to the formation of cancer stem-like cells. More importantly, endogenous c-kit has been shown to be expressed on the cell surface of putative adult cardiac stem cells, and hemangioblasts [40]. Both endogenous c-kit and CD44 are expressed in ovarian cancer cells and ovarian cancer tissues of patients and mice, and to be candidate cell surface markers for ovarian tumor progenitors[41]. Endogenous CD117/c-kit was induced in the transdifferentiated EMT sarcoma tissues [42]. Endogenous c-kit has been up-regulated in ovarian cancer cell lines that have a stem cell phenotype [16]. These data show that endogenous or exogenous c-kit overexpression is closely associated with cancer stem-like cells, and there is a close relationship among c-kit, EMT, and CSC.

In our study, we have observed a cooperative action between c-kit and activated oncoprotein Ras in ACC-M/2 cells, resulting in the formation of larger tumors in nude mice relative to c-kit or Ras itself. In line with our observations, Moriyama et al [43] established a novel xenograft model of human gastrointestinal stromal tumor (GIST) in mice, and found that c-kit expression was observed in each passage and both imatinib and sunitinib significantly reduced the size of the xenograft tumor. And Chew et al [44] has shown that induced expression of the oncogenic Kras in adult transgenic fish led to the development of hepatocellular carcinomas. Thus, our findings provide evidence for the ability of c-kit to potentiate the oncogenic effect of activated Ras in ACC tumorigenesis. An earlier study suggests that Ras also induces the expression of other EMT inducer such as Twist1 and SOX4 in cancer cells [45]. A recent work has showed the role of Claudin-1 as a promoter of the EMT via the c-Abl/Raf/Ras/ERK signaling pathway in hepatocellular carcinoma (HCC) [46]. These data support the assumption that EMT inducers and activated Ras are able to exert the cooperative effects to promote even more dramatic characteristics of EMT and cancer progression [47]. However, the mechanisms underlying this phenomenon still remain unknown.

Although the growth of ACC is slow, the long-term prognosis of these patients is poor and the 15 and 20-year survival rates are rather poor at 35–40% and 10% respectively[5, 6], due to the persistence of tumor growth rate, recurrence after initial treatment, perineural invasion, hematogenous spread and invasion to distant and neighboring tissues. Recent studies have linked EMT with ACC. Targeting the EMT-like phenotypes would seem to represent the potential strategies for the development of novel anticancer therapeutics. Our results in this study are in favor of a strong correlation between c-kit and ACC, and hence point to the prospect of using c-kit as a novel biomarker for prognosis and diagnosis of ACC, as well as a potential molecular therapeutic target for ACC.

## MATERIALS AND METHODS

### Cell culture

Two malignant ACC cells lines, ACC-2 and ACC-M, were obtained from the State Key Laboratory of Oral Disease, Sichuan University. Cells were cultured in RPMI 1640 medium (Gibco) supplemented with 10% heat-inactivated FCS (Hyclone), 2 mmol/L L-glutamine, 25 mmol/L HEPES, and 100 units/mL penicillin and streptomycin in a humidified 5% CO_2_ atmosphere.

### TGF-β1 treatment

1×10^3^ cells /well were placed in serum-free medium, starved for 12 h, then induced with TGF-β1 at 10 ng/mL for 0, 1, 2, and 3 days. Culture medium was changed every other day.

### Gleevec treatment

Gleevec, provided by Novartis (Basel, Switzerland), was dissolved in DMSO to a stock concentration of 10 mM and stored at −20°C. Cells were incubated for 12 h with 5 μM Gleevec.

### Antibodies and reagents

The antibodies and reagents are listed in Supplementary Materials and Methods.

### Cloning, lentivirus preparation, and plasmids

A description of procedures are detailed in Supplementary Materials and Methods.

### Immunofluorescence assay

Experiments were carried out as described in Supplementary Materials and Methods.

### Western blot

Standard procedures for immunoblotting are described in Supplementary Materials and Methods.

### Quantitative real-time reverse transcriptase-PCR

A description of procedures are detailed in Supplementary Materials and Methods.

### Cell proliferation

Cells were incubated with 0.5 mg/mL MTT for 4 hours at 37°C. Then supernatant was removed and 150 mg DMSO were added. Optical densities at 490 nm were measured using culture medium as a blank.

### Wound-healing assay

Experiments were carried out as described in Supplementary Materials and Methods.

### Transwell invasion assays

A description of procedures are detailed in Supplementary Materials and Methods.

### Mammosphere formation assays

Single cells were plated at 10,000 cells/mL on 6-well ultra-low attachment plates (Corning) in serum-free DMEM/F12 supplemented with 20 ng/mL bFGF, 20 ng/mL EGF, 4 mg/mL insulin, 4 mg/mL heparin, 1 mg/mL hydrocortisone, 0.4% BSA and B27. Fresh medium was supplemented every 3 days. The mammospheres were counted at day 14.

### Flow cytometry

A total of 1×10^6^ cells were resuspended in 100 mL PBS containing 2% FBS (FACS buffer), and then incubated on ice for 10 minutes. CD133-APC and CD44-PE (BD Biosciences) were added to cell suspension and incubated on ice for 30 minutes. Cells were washed and resuspended in 500 mL FACS buffer and analyzed using a FACS Calibur Flow Cytometer (Cytomic FC500, Beckman).

### Luciferase reporter assay

The protocol is described in Supplementary Materials and Methods.

### Patients and specimens

One hundred and twenty-one patients with ACC of salivary gland who underwent resection of their tumors without preoperative chemotherapy, hormone therapy or radiotherapy at the Department of Oral and Maxillofacial Surgery, West China Hospital of Stomatology, Sichuan University between 1996 and 2005 were recruited for the study after giving informed consent. Demographic and other variables including primary tumor site, dates of diagnoses, and perineural invasion, local regional recurrence and distant metastasis were retrieved from the database provided by the tumor registry (Table S3). The protocol of the study was approved by the Institutional Ethics Committee of the West China Medical Center, Sichuan University, China. The average follow-up time of all of the patients was 142 months (range 4-192 months). In addition, 10 samples of human normal glands of salivary benign tumors were included in this study.

### Immunohistochemistry

Standard procedures for immunoblotting are described in Supplementary Materials and Methods.

### Xenograft mouse experiments

A total of 5×10^5^ cells in 100 mL PBS were injected subcutaneously into 6-week-old female nude mice. Five mice per group were used in each experiment. Tumor size was monitored by measuring diameters using vernier caliper weekly, and was calculated as πls^2^/6, where l = long side and s = short side as described previously [48]. Tumors were harvested at the 7th week. All animal experiments were approved by the Animal Care Committee of West China Medical Central of Sichuan University, China.

### Statistical analysis

Data are presented as mean±SD. The Student *t* test (2-tailed) was used to determine statistically the significance of differences between groups. Fisher's exact test was used to analyse the association between c-kit and Slug expression and clinicopathological variables. Overall survival curves were estimated using the Kaplan-Meier method, and differences between groups were compared using the log-rank test. Statistical analysis was carried out using the SPSS13.0 software. Probabilities of less than 0.05 were accepted as significance.

## SUPPLEMENTARY MATERIALS TABLES AND FIGURES






