# Supplementary material for: C-kit induces epithelial-mesenchymal transition and contributes to salivary adenoid cystic cancer progression
Source: Oncotarget. 2014 Jan 6;5(6):1491–501. doi: 10.18632/oncotarget.1606 (PMC4039226; doi:10.18632/oncotarget.1606)
Supplement: Supplementary file 1 [file oncotarget-05-1491-s001.pdf]

C-kit induces epithelial–mesenchymal transition and contributes to salivary adenoid cystic cancer progression – Tang et al

Supplementary Materials and Methods.

Antibodies and reagents

Antibodies against N-cadherin, β-catenin, Vimentin, Fibronectin (BD Biosciences), E-cadherin, p-Smad2 (Cell Signaling Technology), c-kit (Abcam), Occludin (Zymed Laboratories), Snail (Abcam), β-actin (Sigma) and Ras (Millipore) were used. Recombinant human TGF-β1 proteins were purchased from R&D Systems. TGFβRI inhibitor SB431542 were purchased from Sigma.

Cloning, lentivirus preparation, and plasmids

The Lenti-X shRNA expression system (Clontech) was used for the construction of the lentiviral expression construct according to the manufacturer's instructions. Short pairs of sense and antisense DNA oligo encoding a sense-loop-antisense sequence to c-kit genes were synthesized for the validated corresponding siRNAs, and sequences are listed in Supplementary Table S1.

The full-length c-kit cDNA was cloned into the pcDNA3 plasmid vector and transfected into cells by LipofectAMINE reagent (Invitrogen) according to the manufacturer's instructions. Stable transfected cells were selected in 400 μg/mL Geneticin and further subcloned.

Immunofluorescence assay

ACC cells were seeded onto coverslips at a density of 10^4/mL and cultured in a 6-well culture plate for 24 hours. Cells grown on coverslips were washed in cold PBS and fixed in 2% paraformaldehyde-PBS for 20 minutes, permeabilized in 0.5% Triton X-100 in PBS for 10 minutes at 4°C, and blocked in 1% bovine serum albumin for 30 minutes at room temperature. Coverslips were incubated overnight with primary antibodies, followed by incubation with TRITC-conjugated secondary antibodies for 1 h, and then stained with DAPI. Finally, coverslips were observed under a fluorescence microscope (Olympus BX51).

Western blot

Total proteins were isolated from the cultured monolayer cells with a total protein extraction kit (Keygen), and protein concentrations were detected by a bicinchoninic acid protein assay kit (Pierce). Thirty-microgram proteins from each sample were separated on 8% SDS-PAGE and transferred electrophoretically to polyvinylidene difluoride membranes (Millipore). Membranes were blocked with 2% bovine serum albumin in TBS containing 0.1% Tween20 (TBST) at 37°C for 2 hours and then incubated for 2 hours respectively with primary antibody. Horseradish peroxidase–conjugated antimouse or antirabbit IgG were used as secondary antibody. Bands were scanned using a densitometer (GS-700, Bio-Rad Laboratories), and quantification was done using Quantity One 4.4.0 software.

Quantitative real-time reverse transcriptase-PCR

Total RNA was isolated with TRIzol reagent (Invitrogen) and treated with RNase-free DNase I (Takara) to avoid genomic DNA contamination. PCR amplification of the cDNA template was done using Thunderbird SYBR qPCR mix (TOYOBO) on ABI PRISM 7300 sequence detection system (Applied Biosystems). Reactions were run in triplicate, and results were averaged. Each value was normalized to GAPDH as the housekeeping gene to control for variations in the amount of input cDNA. Supplementary Table S2 shows the sequences of PCR primers used in this study. The relative expression level of the genes was calculated using the ΔΔCt method.

Wound-healing assay

Cells were plated in 6-well plates at 2.0×10^{5} cells/well. When cells reached 80% confluence, the individual wells were wounded by scratching with a pipette tip and incubated with medium containing no FBS to 0,18 h. Cells were photographed under phase-contrast microscopy (×100) as previously described.

Transwell invasion assays

In vitro cell invasion assays were performed with QCM™ 96-well cell invasion assay kit (Chemicon International, Temecula, CA, USA). 5×10^{4} cells were seeded into the top chamber coated with Matrigel (BD Biosciences). Complete medium was added to the bottom wells to stimulate invasion. After cells were incubated for 24-48 h, they were stained with 0.1% Crystal Violet. The cells that had invaded through matrigel and reached to the reverse side were counted under a microscope in five pre-determined fields at a magnification of ×400. Each assay was performed in triplicate.

Luciferase reporter assay

Cells were transfected with pE-cadherin-luc vector (Clontech) and pRL-TK Vector (Promega) using the Lipofectamine 2000 (Invitrogen), and then were lysed and assayed using the Dual-Luciferase reporter assay system (Promega). Luciferase activity was measured in a luminometer. Results are expressed as relative E-cadherin activity compared with controls after normalizing to Renilla luciferase activity.

Immunohistochemistry

IHC was performed on 4-mm-cut representative sections by the streptavidin-peroxidase method followed as previously described. The rabbit polyclonal antibody against c-kit (ab5506, Abcam, 1:50) and the rabbit polyclonal antibody against Slug (ab27568, Abcam, 1:200) were used for the detection c-kit and Slug in the cancer cells, respectively. The ratio of c-kit and Slug positive cells was counted as the percentage of 1000 tumor cells within 4–6 microscopic fields at×200 magnification irrespective of staining intensity, semiquantitatively graded as follows: negative (0–9%), positive (>10%).
