# Supplementary material for: C-kit induces epithelial-mesenchymal transition and contributes to salivary adenoid cystic cancer progression
Source: Oncotarget. 2014 Jan 6;5(6):1491–501. doi: 10.18632/oncotarget.1606 (PMC4039226; doi:10.18632/oncotarget.1606)
Supplement: Supplementary file 2 [file oncotarget-05-1491-s002.pdf]

C-kit induces epithelial–mesenchymal transition and contributes to salivary adenoid cystic cancer progression – Tang et al

Table S1 shRNA sequences of c-kit

Gene	Sequence(5'-3')
c-kit	5'-GATCCGtctagccagagacatcaagTTCAAGAGActtgatgtctctggctag aTTTTTTACGCGTG-3'
c-kit Negative	5'-GATCCGtcagcacatggattcgcaaTTCAAGAGAttgcgaatccatgtgctg aTTTTTTACGCGTG-3'

Table S2 Gene primers of RT-PCR

Gene	Forward primer (5'-3')	Revese primer (5'-3')
c-kit	ATTTTCTCTGCGTTCTGCTCCTAC	CGCCCACGCGGACTATTA
Slug	TGATGAAGAGGAAAGACTACAG	GCTCACATATTCCTTGTCACAG
Snail1	GCTCCACAAGCACCAAGAGT	CATGGCAGTGAGAAGGATGT
E-cadherin	CCCATCAGCTGCCCAGAAAATGAA	CTGTCACCTTCAGCCATCCTGTTT
N-cadherin	CCGACGAATGGATGAAAGACC	TTGCAGCCTATGCCAAAGC
Vimentin	GAGAACTTTGCCGTTGAAGC	GCTTCCTGTAGGTGGCAATC
TGF-β1	CCAACTATTGCTTCAGCTCCA	TTATGCTGGTTGTACAGGG
ZEB1	TGCACTGAGTGTGGAAAAGC	TGGTGATGCTGAAAGAGACG
ZEB2	CGCTTGACATCACTGAAGGA	CTTGCCACACTCTGTGCATT
Prrx1	GGATCCACCATGACCTCCAGCTACGGGCA	GAATTCTCAGTTGACTGTTGGCACCT
HOXB7	CCAGCCTCAAGTTCGGTTTTC	CCCGAACCCGCTCCATAG
GAPDH	ATGACCCCTTCATTGACCTCA	GAGATGATGACCCTTTTGGCT

Table S3 Clinicopathological features of the ACC patients (*n*=121)

Clinicopathological features	No. of cases
Age(years) at diagnosis	121
<50	45
>=50	76
Sex	121
Female	67
Male	54
Complaints, months	121
<12	64
≥12	57
Site	121
Minor salivary gland	63
Major salivary gland	58
Histological subtype	121
Tubular/ Cribiform	85
Solid	36
Resection margins	121
Free	88
Affected	33
TNM stage	121
Ⅰ-Ⅱ	34
Ⅲ-Ⅳ	87
Perineural invasion	100
Yes	47
No	53
Local regional recurrence	121
Yes	30
No	91
Distant metastasis	103
Yes	39
No	64
