# Supplementary material for: C-kit induces epithelial-mesenchymal transition and contributes to salivary adenoid cystic cancer progression
Source: Oncotarget. 2014 Jan 6;5(6):1491–501. doi: 10.18632/oncotarget.1606 (PMC4039226; doi:10.18632/oncotarget.1606)
Supplement: Supplementary file 3 [file oncotarget-05-1491-s003.pdf]

C-kit induces epithelial–mesenchymal transition and contributes to salivary adenoid cystic cancer progression – Tang et al

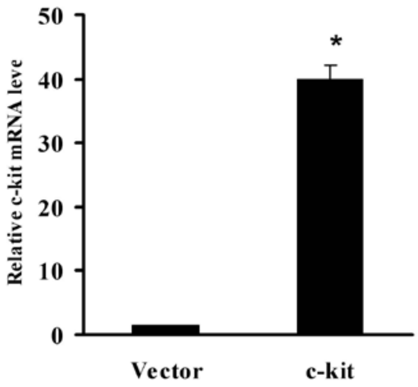

Figure S1: The c-kit mRNA level in ACC-M cells. The c-kit mRNA level was quantified by real-time PCR (*$p<0.001$, compared to the control).

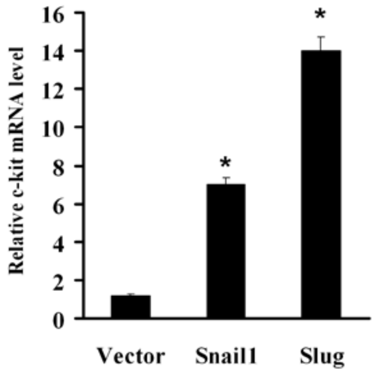

Figure S2: Induction of c-kit by other EMT-inducers. Expression of c-kit mRNA in ACC-M cells ectopically expressing either empty vector or the indicated EMT-inducing genes was assessed by real-time PCR (*$p<0.001$, compared to the control).

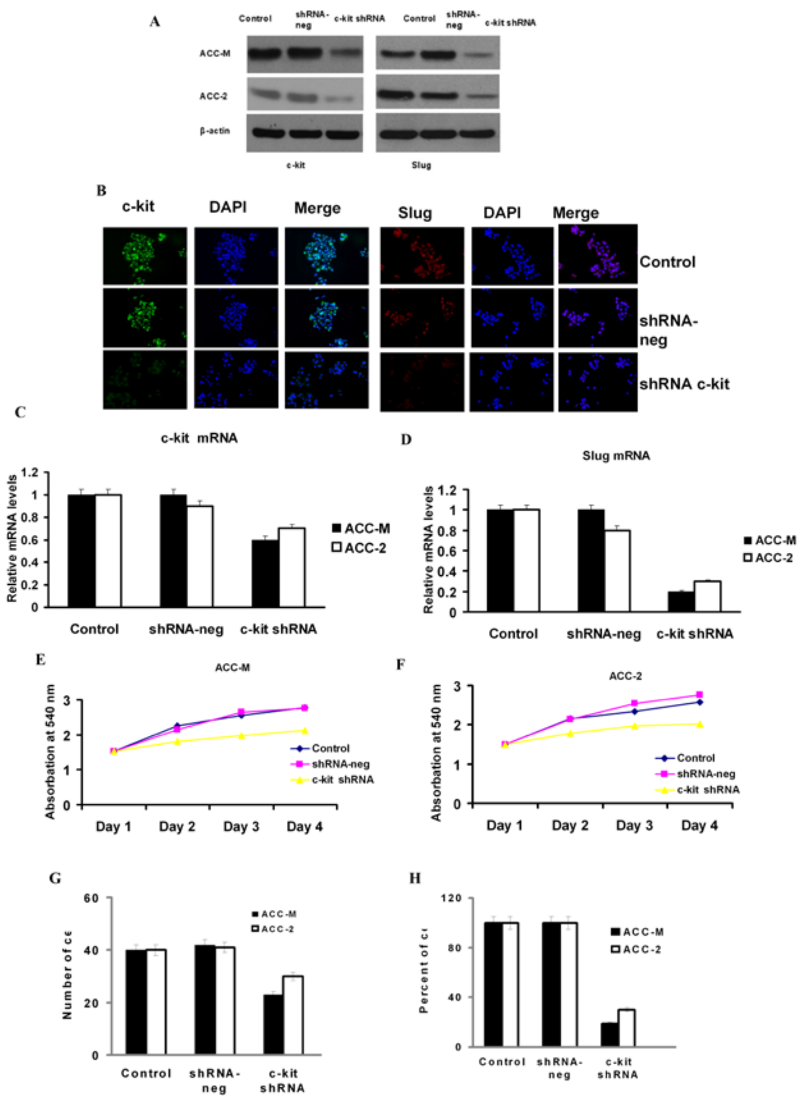

Figure S3: Silencing of c-kit in ACC-M and ACC-2 cells inhibited their invasive ability. A), Expression of c-kit and Slug protein after c-kit silencing in ACC-M and ACC-2 cells. B), Immunofluorescence of c-kit and Slug after c-kit silencing in ACC-M and ACC-2 cells. Scale bar =100μm. C and D), Expression of c-kit and Slug mRNA after c-kit silencing in ACC-M and ACC-2 cells. E and F), Proliferation of ACC-M cells (Left) and ACC-2 cells (Right) expressing control, shRNA-neg and shRNA-c-kit were measured using MTT assays. G and H), Invasion assay of ACC-M and ACC-2 cells upon shRNA transfection. The mean was derived from cell counts of five fields, and each experiment was repeated at least three times (*p<0.001, compared to the control).

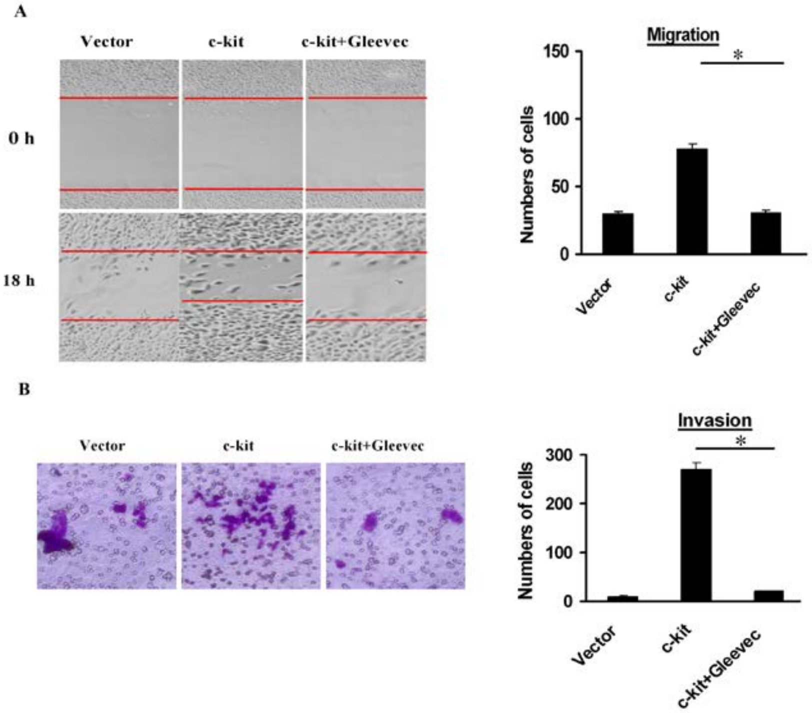

Figure S4: Gleevec had reversal of the enhanced migration and invasion ability of ACC-M cells with c-kit overexpression. A and B), Migration (A) and invasion (B) assays in stable ACC-M cells. The mean was derived from cell counts of 5 fields, and each experiment was repeated 3 times (* $P < 0.001$, compared with the c-kit overexpression group). Representative images of migrated and invaded cells are shown.

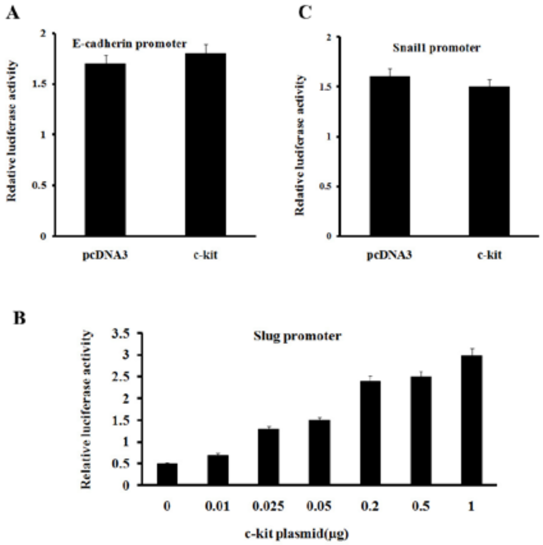

Figure S5: c-kit indirectly regulated E-cadherin transcription. A), 293T cells were transiently co-transfected with 1 μg of c-kit expression construct or 1 μg of pcDNA3, together with E-cadherin promoters. Relative luciferase activity is shown. Error bars represent mean ± SD of triplicate experiments. B), 293T cells were transiently co-transfected with the indicated amounts of c-kit expression vectors (1 μg of the empty

vector was used in column 1)and Snail1 promoter reporter. Relative luciferase activity is shown. Error bars represent mean ± SD of triplicate experiments. C) ,293T cells were transiently co-transfected with 1 µg of c-kit expression construct or 1 µg of pcDNA3, together with Slug promoter. Relative luciferase activity is shown. Error bars represent mean ± SD of triplicate experiments.

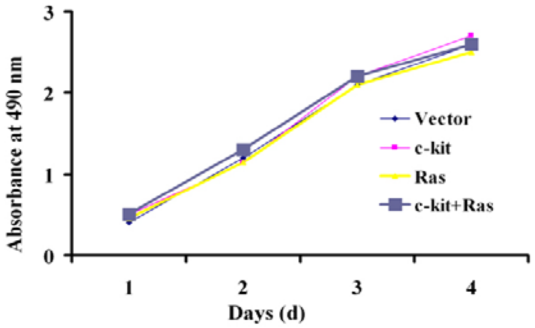

Figure S6: Proliferation of ACC-M cells expressing vector, c-kit, Ras and c-kit +Ras were measured using MTT assays. All the cells exhibited roughly the same growth rates with on statically significant differences.

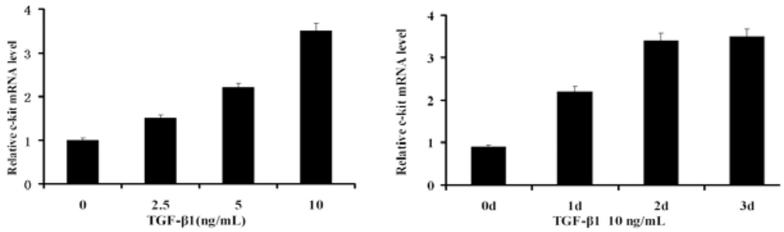

Figure S7: Relative c-kit mRNA expression levels in ACC-M cells treated with activated TGF-β1 at indicated time and concentrations. Error bars represent mean ± SD of triplicate assays.
